# Are Transcription Factors Plausible Oncotargets for Triple Negative Breast Cancers?

**DOI:** 10.3390/cancers14051101

**Published:** 2022-02-22

**Authors:** Marta Marqués, Maria Alba Sorolla, Izaskun Urdanibia, Eva Parisi, Iván Hidalgo, Serafín Morales, Antonieta Salud, Anabel Sorolla

**Affiliations:** 1 Research Group of Cancer Biomarkers, Lleida Institute for Biomedical Research Dr. Pifarré Foundation (IRBLleida), Av. Alcalde Rovira Roure, 80, 25198 Lleida, Spain; mmarques@irblleida.cat (M.M.); msorolla@irblleida.cat (M.A.S.); iurdanibia@irblleida.cat (I.U.); eparisi@irblleida.cat (E.P.); ihidalgo@irblleida.cat (I.H.); serafinmorales01@gmail.com (S.M.); asaluds@hotmail.com (A.S.); 2 Department of Medicine, University of Lleida, Av. Alcalde Rovira Roure, 80, 25198 Lleida, Spain; 3 Department of Medical Oncology, Arnau de Vilanova University Hospital (HUAV), Av. Alcalde Rovira Roure, 80, 25198 Lleida, Spain

**Keywords:** breast cancer, transcription factors, cancer progression, cancer initiation, prognosis

## Abstract

**Simple Summary:**

Triple negative breast cancer is a type of breast cancer that does not have a selective and effective therapy. It is known that this cancer possesses high abundance of certain proteins called transcription factors, which are essential for their growth. However, inhibiting transcription factors is very difficult with common therapeutics due to their inaccessibility inside the cell and their molecular structure. In this work, we identified the most important transcription factors for the growth of triple negative breast cancers, and that can predict worse clinical outcome. Moreover, we described different strategies that have been utilised to inhibit them. A successful inhibition of these transcription factors could reduce the mortality and convalescence associated with triple negative breast cancers.

**Abstract:**

Breast cancer (BC) is the most diagnosed cancer worldwide and one of the main causes of cancer deaths. BC is a heterogeneous disease composed of different BC intrinsic subtypes such as triple-negative BC (TNBC), which is one of the most aggressive subtypes and which lacks a targeted therapy. Recent comprehensive analyses across cell types and cancer types have outlined a vast network of protein–protein associations between transcription factors (TFs). Not surprisingly, protein–protein networks central to oncogenesis and disease progression are highly altered during TNBC pathogenesis and are responsible for the activation of oncogenic programs, such as uncontrollable proliferation, epithelial-to-mesenchymal transition (EMT) and stemness. From the therapeutic viewpoint, inhibiting the interactions between TFs represents a very significant challenge, as the contact surfaces of TFs are relatively large and featureless. However, promising tools have emerged to offer a solution to the targeting problem. At the clinical level, some TF possess diagnostic and prognostic value in TNBC. In this review, we outline the recent advances in TFs relevant to TNBC growth and progression. Moreover, we highlight different targeting approaches to inhibit these TFs. Furthermore, the validity of such TFs as clinical biomarkers has been explored. Finally, we discuss how research is likely to evolve in the field.

## 1. Introduction

**Figure 1:**
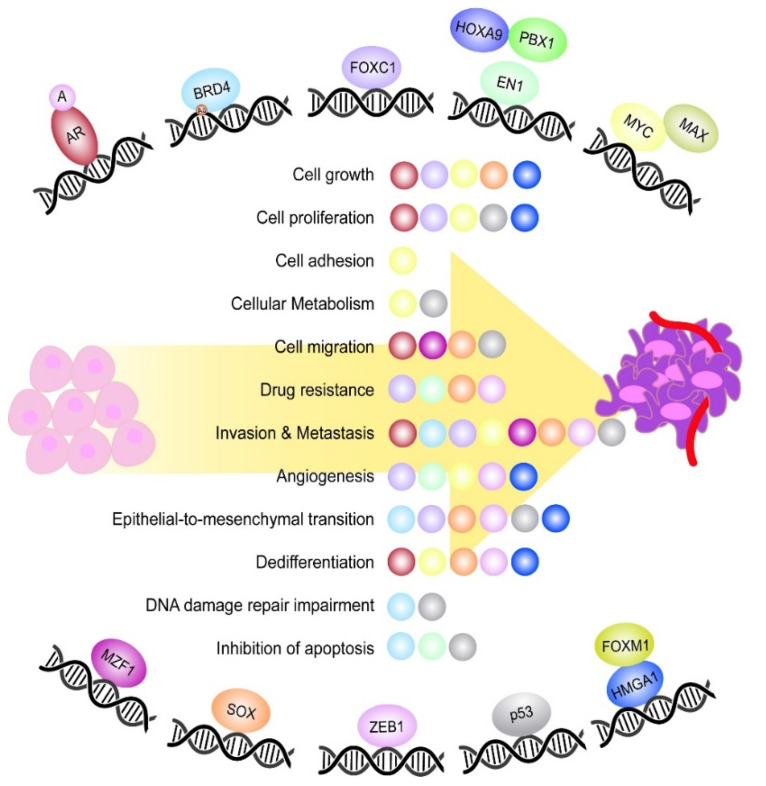
Most relevant TFs playing a role during TNBC tumorigenesis. Schematic representation of the most relevant TFs controlling different aspects of TNBC tumorigenesis. The TFs EN1, MYC and HMGA1 are represented together with their respective binding partners, which constitute an active DNA binding complex. Moreover, the TF BRD4 is featured with an acetyl group (Ac), which represents the DNA epigenetic modification undergone by the TF. In the middle, it is shown a list of the main oncogenic processes controlled by the TFs during TNBC onset and progression. Each ball represents the TF involved in the process and is the same colour as the TF.

**Figure 2:**
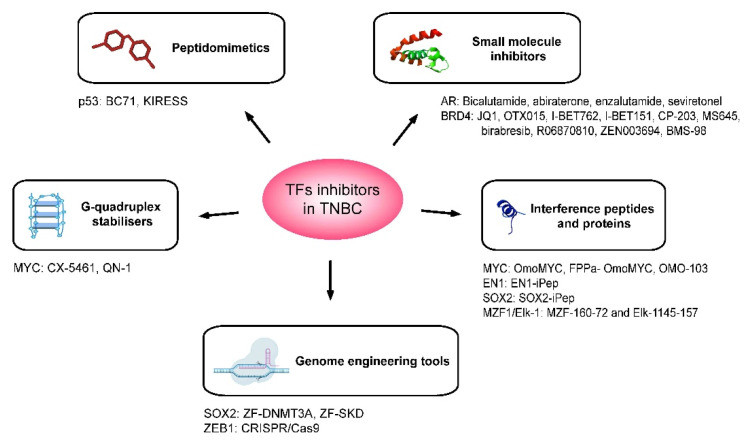
Therapeutic agents that have been developed for the inhibition of oncogenic TFs in the context of TNBC. Schematic representation of the five main strategies adopted for the inhibition of TFs having a role in TNBC tumorigenesis. These are small molecule inhibitors, interference peptides and proteins, peptidomimetics, G-quadruplex stabilisers and genome engineering tools. The target of the transcription factor and the names of inhibitors are specified below for each type of therapeutic agent.

Breast cancer (BC) was the most diagnosed malignancy in women and the fifth leading cause of cancer deaths worldwide in 2020 [1]. BCs can be classified according to the presence and absence of oestrogen receptor (ER), progesterone receptor (PR) and human epidermal growth factor receptor 2 (HER2) or ERBB2. Triple negative breast cancer (TNBC) lacks all three receptors and represent 15–20% of all breast carcinomas [2]. TNBC is the most challenging and difficult-to-treat BC subtype due to unsuitability of anti-ER, anti-PR and anti-ERBB2 treatments, and lacks a targeted therapy. Despite all the chemotherapies available to treat these tumours, they still show a risk of recurrence and mortality rate within five years of the diagnosis of 50 and 37%, respectively [3,4]. One of the reasons is the emergence of multi-drug resistance mechanisms due to oncogenic transcription factors (TFs) overexpression, stem cell (SC) selection and immune surveillance escape [5,6,7]. TFs, through protein–protein interactions with their binding partners and the DNA, can initiate and maintain essential transcription programs for oncogenesis. We have identified ten TFs as the most relevant in TNBC. These are the androgen receptor (AR) [8], bromodomain 4 (BRD4) [9], forkhead box C1 (FOXC1) [10], homeobox protein engrailed-1 (EN1) [11], MYC [12], myeloid zinc finger (MZF1) [13], p53 [14], sex-determining region Y-related HMG-Box (SOX) members [15], zinc finger E-box binding homeobox 1 (ZEB1) [16] and high mobility group AT-hook 1 (HMGA1) [17]. They are major controllers and final effectors of multiple facets of TNBC progression, such as dedifferentiation, chemoresistance, epithelial-mesenchymal transition (EMT) and metastatic dormancy [18] (Figure 1). However, their effective inhibition has remained elusive due to the lack of binding pockets and intracellular localisation [19]. Many efforts have been made to achieve TFs inhibition in TNBC, which include the use of small inhibitors, peptides, proteins, peptidomimetics, G-quadruplexes and, more recently, genome editing tools [19,20,21,22] (Figure 2) (Table 1 and Table 2). Due to their prominent role in cancer progression and selective overexpression in TNBC, some TFs have been proposed as valuable biomarkers of diagnosis, stratification and prognosis.

In this review, we provide an overview of the research on recent advances in TFs relevant to TNBC initiation and progression. Moreover, we highlight different therapeutic approaches used to inhibit these TFs, as well as the different TFs that have been investigated as clinical biomarkers for TNBC. Finally, we discuss how research is likely to evolve in the field. 

## 2. TFs Having a Role in TNBC Progression

### 2.1. AR

AR, also known as nuclear receptor subfamily 3, group C, member 4 (NR3C4) is a steroid hormone nuclear receptor. Structurally, AR has a N-terminal domain (NTD), DNA binding domain (DBD) and C-terminal ligand binding domain (LBD). The DBD is highly conserved and enriched in cysteines, and contains two zing fingers, one responsible for direct DNA binding and the other for head-to-head receptor dimerisation [8]. Upon binding of androgen ligands to AR, AR dimerises and translocates to the nucleous in order to undergo its DNA-binding activity [56]. AR is expressed in 10–43% of TNBC [57]. It has been shown that enzalutamide, an AR antagonist, reduces cell proliferation, anchorage-independent growth, migration, and invasion in TNBC xenografts [23]. In another work, the authors found that AR is upregulated in forced anchorage-independent conditions and that enzalutamide treatment diminishes the cancer stem cell (CSC) population in vitro and in vivo [24]. Additionally, single-cell sequencing analyses discovered that *AR* transcripts are significantly more expressed in circulating tumour cells than in primary tumour cells from TNBC patient-derived xenografts (PDXs) [58]. Altogether this suggests that AR signalling could be involved in metastasis and recurrence of these tumours.

### 2.2. BRD4

BRD4 is a member of the bromodomain and extra-terminal (BET) domain protein family. BET members are acetyl-lysine readers, which bind to acetylated chromatin and TFs; they can modulate transcriptional programs such as those involved in cell cycle control and proliferation [9]. BRD4 contains two N-terminal bromodomains called BD1 and BD2 and the C-terminal domain ET. 

BRD4 has been found to be altered in 12% of BCs. Of this percentage, 0.55% corresponds to mutations, 0.37% to gene fusions, 0.65% to amplifications, 6.46% to high mRNA levels, 2.21% to low mRNA and 1.75% to other alterations [59]. This status is independent of the BC type. BRD4 is overexpressed or amplified in TNBC, highlighting its importance in the progression of these cancers [60]. Shi and colleagues discovered a novel mechanism by which BRD4 promotes tumorigenesis in basal-like BCs consisting in the interaction between BRD4 and deacetylated Twist, a key factor of the EMT program, which directs *WNT5A* expression [25]. Disruption of such an interaction by drugs successfully suppressed *WNT5A* expression and, consequently, the malignant phenotype in SUM1315 cells and xenografts [25]. In contrast, as demonstrated in polymerase chain reaction-based (PCR) array and the immunodetection of EMT markers, BRD4 blocks the EMT program in the TNBC cell lines MDA-MB-231 and SUM149PT [26].

Another association partner of BRD4 is Polycomb repressive complex 1 (RING1B), as demonstrated in a study in TNBC cells MDA-MB-231 [27]. RING1B-depleted MDA-MB-231 cells become less metastatic in vivo, supporting a pro-tumorigenic role of the complex BRD4-RING1B in these tumours. Pharmacologic inhibition of BRD4 by a panel of inhibitors of bromodomain and extra-terminal proteins (BETi) preferentially killed TNBC cells [28]. In addition, treatment with the cell-permeable small molecule JQ1, a member of the BETi family, induced apoptosis, cell senescence, basal-to-luminal differentiation and decreased in vivo TNBC tumour growth [28,29]. JQ1 and GSK525762A have also been reported to reduce BRCA1 levels in TNBC cells hampering homologous recombination-mediated DNA repair [30]. Moreover, JQ1 and VS-6063 (an inhibitor of the focal adhesion kinase (FAK))-mediated co-inhibition of BRD4/MYC and FAK cooperatively induced apoptosis in TNBC cells and reduced in vivo tumour growth in the TNBC syngeneic model 4T1 [31].

### 2.3. FOXC1

The forkhead box C1 TF (FOXC1) belongs to the forkhead family of TFs. Its structure contains one N-terminal and one C-terminal transactivation domains and a transcription inhibitor domain [10]. FOXC1 is implicated in the formation of vasculature and organ development during embryogenesis. It is also involved in cell growth, metabolism regulation and longevity [61].

*FOXC1* has been found to be highly overexpressed in basal-like BCs compared to other BCs [62,63,64]. Besides, the presence of FOXC1 is characteristic of immuno-suppressed TNBC in a further subclassification of these tumours [65]. 

Regarding its biological role in TNBC, it has been suggested FOXC1 to be a critical player in different tumorigenic processes, such as the induction of survival, proliferation, EMT, metastasis, invasiveness and chemoresistance [10,61,66,67,68]. These are undertaken through the activation of different signalling pathways, such as epidermal growth factor receptor (EGFR)/FOXC1/Nuclear factor κB (NFκB), Phosphatidylinositol-3-kinase (PI3K)/protein kinase B (AKT)/Mammalian target of rapamycin (mTOR) and wingless-type MMTV integration site family, member 5A (WNT5A)/NFκB/matrix metallopeptidase 7 (MMP7) axis. The work of Huang et al. supports the same critical involvement of FOXC1 in TNBC progression since the CRISPR/Cas9-mediated deletion of FOXC1 super-enhancer hampers 3D growth and clonogenic growth in cells and xenografts [69]. In addition, these authors performed a complex bioinformatic analysis and identified FOXC1 as the most significant regulator of invasion and metastasis [69]. 

### 2.4. EN1

The homeobox protein engrailed-1 (EN1) belongs to the homeodomain family of TFs. The family members are characterised by a helix-turn-helix DNA-binding motif known as homeodomain or homeobox [11]. The homeobox is a conserved 60 amino acid sequence (homeobox) composed of three alpha helices. The DNA recognition function is given by the union of the C-terminal helix (third helix) to the major groove. The N-terminal helix aligns to the minor groove. In neural progenitor cells, EN1 is responsible for expanding and maintaining the pool of dopaminergic neurons with prosurvival activity, in order to guarantee the correct development of the central nervous system (CNS) [5]. EN1 presence protects neurons from apoptotic insults, and EN1 downregulation causes dopaminergic neuronal degeneration, a hallmark of Parkinson’s disease [70,71]. 

EN1 is selectively overexpressed in TNBC tumours, either basal-like BCs or quintuple negative BCs (ER-, PR-, HER2-, Cytokeratin 5/6 (CK5/6) and EGFR-) [5,6,72]. *EN1* inhibition via shRNA results in G1 arrest and in an increase of apoptosis in basal-like BC cells [5]. In addition, EN1 is involved in activating prosurvival pathways, rendering cells more resistant to chemotherapy [5]. In addition to that, EN1 can activate formation and maturation of new blood vessels, with its consequent higher risk of tumour dissemination [6]. 

### 2.5. MYC

MYC is a proto-oncogene, member of the basic region helix-loop-helix leucine zipper (bHLHZip) family, therefore, a nuclear-DNA binding protein [12]. MYC is a regulator of ~15% of the genome and plays an essential role in normal and cancer cells, promoting cell proliferation, growth, adhesion, metabolism, angiogenesis, differentiation and apoptosis [19]. MYC is a participant in a high number of pathways, among them, the p53 pathway the depletion or mutation of which can induce MYC constitutive activation [73]. MYC requires an interaction with MAX (bHLHZip protein) for its transcriptional and transforming activity [12]. Locus amplification and overexpression is described in many human malignancies due to variations in MYC promoter activity and protein stability. In mammary cells, expression of MYC is normal and MYC over-activation in BC is responsible for maintaining and expanding the pool of MaSC (mammary stem cells) leading to an increase in the SC phenotype [73]. One study shows that 45% of *BRCA1*-mutated tumours possess *MYC* amplification and that *BRCA1* mutations are typical in TNBC [74]. 

Cancer cells possess greater MYC dependence than normal cells, but it is not clear how MYC regulates cancer transition. Although it has been shown that MYC controls many different cellular functions necessary for successful invasion, translocation, seeding, and growth at distant sites, MYC overexpression by itself could not induce invasion in normal-like breast cells in vitro, suggesting that cooperation with other pathways is needed [75]. 

### 2.6. MZF1

MZF1 is a TF that belongs to the Krüppel family of zinc fingers [13]. The human full-length isoform contains a highly conserved SCAN, transactivation domain (TAD) and 13 Krüppel-like zinc finger motifs. When firstly studied in the hematopoietic compartment, it was found that MZF1 controlled cell proliferation acting as tumour suppressor in hematopoietic cells [13]. In contrast, MZF1 acts as oncogene in many solid cancers such as breast, cervical, colorectal, liver, lung and prostate cancer [76]. MZF1 is overexpressed in the TNBC cell lines Hs578T and MDA-MB-231 correlating with higher migration and invasion capabilities, which seems to be mediated by the inactivation of the insulin growth factor 1 receptor (IGF1R) by MZF1 at the promoter level [77]. Interestingly, MZF1 contains an acidic domain that is necessary for protein–protein interaction with the heparin domain of Ets-like protein-1 (Elk-1). When such interactions occur, the heterodimer binds to the protein kinase C (PKCα) promoter region, activating PKCα. PKCα expression is a crucial regulatory step in the EMT process, in the development of breast CSCs, favouring tumour growth, recurrence and metastasis [43]. Elk-1/MZF1 and PKCα overexpression have been correlated with decreased survival outcomes in TNBC due to the acquisition of higher migration and invasion capacities [43].

Regarding gene expression analysis, one study unveiled a core of TFs having a role in TNBC progression [78]. The core contains MZF1 and other four TFs: SOX10, ZEB1, ETS1 and GATA2, which controls genes associated to EMT and CSC. The presence of MZF1, SOX10 and ZEB1 help to distinguish very precisely between TNBC and non-TNBC cell lines [78]. 

### 2.7. SOX Members

The SRY (sex-determining region Y) homology box SOX family of TFs regulates cell fate during embryonic development [15]. This family has DNA binding function, since it contains high mobility group (HMG) box, which is highly conserved [79]. To date, 20 different *Sox* genes have been identified and classified in eight different groups according to their gene sequence and domain structure [80]. SOX factors are expressed in different stages of mammary development and in different mammary cell progenitors orchestrating mammary stem cell fate. For instance, SOX9 and SOX10 are expressed in foetal mammary stem cells [81,82,83], ER- luminal progenitors and basal cells. SOX11 is expressed in embryonic mammary epithelial cells [84] and SOX4 in postnatal basal cells [81]. 

In normal tissues, the SOX transcriptional program is tightly controlled to cover the needs related to tissue homeostasis and tissue repair. However, in cancer, there is an aberrant activation of the SOX program, which increases tumoral heterogeneity, leading to the acquisition of therapy resistance and activation of oncogenic processes such as EMT. High levels of SOX10 upregulates genes associated with EMT and confers invasive capabilities to mammary organoids [85] and mesenchymal-like phenotype to murine mammary tumours [81]. Similarly, both SOX9 and SOX11 enhance the ability of BC cells (MCF7 cells carrying the oncogene *v-rasH*, namely MCF7ras cells) to metastasise to the lungs [86], together with Slug, and the ability of DCIS.com cells to colonise the bone and the brain when injected via tail vein, respectively [83,87]. The DCIS.com cell line is a ductal carcinoma in situ BC cell line that forms DCIS-like lesions in in vivo mouse models, similar to primary human DCIS lesions. Moreover, SOX4 overexpression increases migration and invasion in normal-like breast cells MCF10A through the activation of EMT and transforming growth factor beta (TGF-β) [88]. Targeted inhibition of SOX2 with artificial TFs reduces the growth of MCF7 xenografts [47].

The fact that SOX members govern stem-cell fate has consequences in cancer, such as the acquisition of stem-like properties in tumours, which are responsible for therapy resistance and aggressiveness. Notably, Rodriguez-Pinilla et al. found SOX expression in 43% of basal-like BCs, more than in any other BC subtype [89]. Several SOX members have been shown to be involved in resistance to anti-hormonal agents. In particular, SOX9 upregulation has been detected in tamoxifen-resistant MCF7 and T47D cells [90]. Similarly, tamoxifen-resistant MCF7 cells display high levels of SOX2 and SOX11 [91,92]. Likewise, downregulation of SOX9 and SOX2 sensitises BT-474 and tamoxifen-resistant T47D cells to tamoxifen [91]. In addition, downregulation of SOX11 confers sensitivity to tamoxifen in tamoxifen-resistant MCF7 cells [92]. 

### 2.8. ZEB1

ZEB1, also known as TCF8 or DeltaEF1, is a zinc finger TF that belongs to the homeodomain family of TFs. ZEB1 has one homeodomain and seven zinc fingers. ZEB1 was firstly discovered to play a role in normal development in chicken [16] and later in mammals [93]. During embryogenesis and tumorigenesis, ZEB1 together with other molecules, controls EMT plasticity between epithelial and mesenchymal states [94]. Such a balance is critical for metastasis [95]. Several mechanisms of ZEB1-induced metastasis in TNBC have been described in the literature. For instance, the long non-coding RNA linc-ZNF469-3, through its interaction with miR-574-5p, affects ZEB1 and favours lung metastasis [96]. Moreover, ZEB1 upregulates circular RNAs ((circ)RNAs) from the WWC3 locus, promoting metastasis through the Ras signalling pathway [97]. Moreover, miR-708-3p targets ZEB1 and hinders EMT [98]. In addition, the type I receptor tyrosine kinase–like orphan receptor (ROR1) stimulates EMT and metastasis, and ROR1 downregulation decreases ZEB1 among other EMT-associated proteins [99]. Furthermore, the RNA polypyrimidine tract-binding protein PTBP3 enhances *ZEB1* mRNA stability by binding to its 3′-UTR, thereby promoting EMT and metastasis [100].

ZEB1 is also involved in other oncogenic-related processes such as inflammation, endothelial transdifferentiation, immune system escape, chemoresistance and stemness [101,102,103]. Regarding stemness, ZEB1 promotes the transition of non-CSC to CSC in basal-like BCs [104]. The *ZEB1* promoter remains, simultaneously, in an active and inactive epigenetic state, tipping the balance towards one or the other depending on the stimuli in the tumour microenvironment [105]. Moreover, it has been shown that Ataxia telangiectasia mutated (ATM) phosphorylates and stabilises ZEB1 in response to DNA damage, promoting radioresistance in TNBC [105]. Furthermore, miR-203, a target of ZEB1, has been shown to sensitise BC cells to the chemotherapeutic drugs gemcitabine and paclitaxel [106].

### 2.9. p53

The tumour suppressor protein, p53 is primarily a TF that is biologically active in its homotetrameric form. The domain structure of p53 consists of a N-terminal transactivation domain (TAD) followed by a proline rich region (PRR), a DNA binding domain, a tetramerisation domain (OD) and a regulatory carboxyl terminus domain (CTD) [14]. *TP53* mutations and/or deletions are highly prevalent in cancer (50%) and correlate with aggressiveness and bad prognosis [73]. There are many mutation variants of *TP53* identified (missense mutations, allele deletions, point mutations, etc), mostly found at one of the five highly evolutionary conserved regions of the protein, including the DNA-binding domain residues. These changes result in a conformationally aberrant protein that misfolds, aggregates, accumulates and inactivates, which ultimately increases the tumorigenic potential in cells [107]. In TNBC/basal-like BC, *TP53* alterations are present in >80% of the tumours, mostly in the form of deletions or insertions. In contrast, only 19% of HR-positive/luminal tumours present *TP53* alterations, which are primarily missense mutations. This evidence supports the contribution of *TP53* to TNBC/basal-like BC onset, which seems to be mostly through loss of tumour suppressive functions rather than oncogenic gain (gain-of-function *TP53* mutations) [108]. 

Traditionally, the role of p53 in cancer has been associated with the promotion of DNA repair, apoptosis, senescence, or cell cycle arrest. Recently, p53 has emerged as a negative regulator of adult stem cell self-renewal in the hematopoietic, neural, and mammary gland systems. Indeed, its loss leads to abnormal expansion of the SC compartment and increases its repopulating ability. p53 maintains the pool of quiescent hematopoietic SCs [73]. Moreover, loss of function of p53 results in the abolition of *TP53*-mediated checkpoints and stress responses, and recent evidence points out a role of microRNAs (miRNAs) in the process [73,108]. This loss appears in all phases of tumorigenesis: initiation, progression, metastasis, and tumour maintenance [73]. Moreover, p53 has been described to regulate the expression of a number of miRNAs that control several biological processes including cell cycle, EMT, cell plasticity, survival and metabolism. p53 can bind to the *MIR30A* promoter and induce the transcription of miRNA strands 5p and 3p. Both miRNAs showed the capacity of targeting *ZEB2*, being involved in EMT, tumour cell migration and drug resistance [108]. Many researchers have shown that there are two premises related to p53. On the one hand, tumour cells with mutant forms of p53 are addicted to this protein, a silencing mutant form that inhibits cell proliferation, and, on the other hand, mutant p53 proteins possess pro-tumorigenic function [109]. There is a frequent single nucleotide polymorphism (SNP) at the amino acid 72 (Pro72Arg) that leads to a gain-of-function activity of p53. This SNP makes p53 able to bind to the peroxisome proliferator-activated receptor γ co-activator 1α (PGC-1α) gene promoter region thereby increasing its expression. This higher expression has an impact on mitochondrial function and so, in cell metabolism and in metastatic capability [109]. Another known mechanism by which p53 mutants confer pro-tumorigenic function is due to the ability of these mutants to bind and inhibit the p53 family members, which themselves possess tumour suppressive function. Furthermore, mutants can bind to and enhance the stability or activity of certain pro-tumorigenic TFs [109]. 

### 2.10. HMGA1

The High mobility group AT-hook 1 (HMGA1) is a small chromatin remodelling protein that regulates gene expression by binding to AT-rich regions of the DNA minor groove. It has been shown that HMGA1 can induce stem-cells features in TNBC and its silencing impairs tumour growth, reverses EMT and in vivo tumorigenesis in MDA-MB-231 orthotopic xenografts [17]. A posterior work demonstrated that HMGA1 interacts with the TF FOXM1 to promote tumour angiogenesis in TNBC through the transcriptional activation of vascular endothelial growth factor A (VEGFA) [110]. Thus, it is suggested that blocking the interaction between HMGA1 and FOXM1 could be an attractive therapeutic approach for TNBC. A recent study performing gene network analysis using the software SWIM has confirmed the cooperation between HMGA1 and FOXM1 together with MYBL2 at contributing to TNBC pathogenesis [111].

## 3. Strategies for Targeting and Inhibiting TFs

Targeting and inhibition of TFs have largely been considered very challenging by conventional therapeutics, such as biologicals and small-molecule inhibitors, due to their intracellular location and the lack of grooves on their contact surfaces [19]. In this section, we will describe different strategies used to target the TFs previously mentioned (Figure 2) (Table 1 and Table 2) in the context of TNBC. Among the TFs targeted, MYC has been the one to which researchers have dedicated more efforts.

### 3.1. Small Molecule Inhibitors

Although the clinical benefit of small inhibitors targeting AR for TNBC treatment is not entirely clear, a few small inhibitors have been developed for this purpose. Examples are the first-generation AR antagonist bicalutamide, or the second-generation inhibitors abiraterone and enzalutamide, which showed modest clinical benefits in clinical trials, not suitable for all AR-positive TNBCs and with no differences in clinical responses between AR-High and AR-Low expressing tumours, respectively [53,54,112,113]. Seviteronel, another AR antagonist, did not provide objective tumour responses in locally advanced or metastatic TNBC patients [55].

Other small inhibitors able to recognise and bind particular TFs motifs are BETi. After being discovered that thienodiazepines could bind BRD4 in 2009, later in 2010, the BET inhibitor (BETi) JQ1 was characterised for the first time as a competitive inhibitor of the acetyl-lysine recognition motif or bromodomain of BRD4. JQ1 is able to displace BRD4 from chromatin and has anti-tumoral activity in nuclear protein in testis (NUT) midline carcinoma (NMC) [114]. Mechanistically, JQ1 has demonstrated to reduce TNBC growth through the disruption of the BRD4-MYC interaction [32] and, independently of it, by blocking the interactions between ATPase-family AAA-domain-containing 2 protein (ATAD2), BRD2, BRD4, and androgen receptor (AR) [33], or by suppressing Aurora kinases A and B [34]. Moreover, synergistic interaction at inducing apoptosis in TNBC cells has been demonstrated between JQ1 and GSK2801, a BAZ2/BRD9 inhibitor [35]. Similarly, synergistic inhibition of cell growth was shown in the MYCN high-expressing TNBC MDA-MB-468 cells treated with JQ1 and the MEK inhibitor trametinib [36]. The same regimen was the most effective in reducing tumour burden in three TNBC PDXs [36]. Another BETi, specific of BRD2, BRD3 and BRD4, named OTX015 or MK-8628, exerted synergistic activity in combination with the mTOR inhibitor everolimus in the TNBC cells HCC1937 and MDA-MB-231, and in MDA-MB-231 xenografts [37]. OXT015 treatment as a single agent decreased MYC protein and mRNA levels in TNBC MDA-MB-468 cells [37,115]. Beyond chemotherapeutics, vitamin C has also been shown to potentiate the anti-tumoral in vitro effect of structurally different BETi, ie. JQ1, I-BET762, I-BET151 and CP1-203 in TNBC cells, and to reduce tumour volumes, lung and liver metastatic lesions in MDA-MB-231 xenografts [38]. The synergy observed is through to the suppression of histone acetylation, which is caused by the upregulation of histone deacetylase 1 expression after vitamin C treatment [38]. This lowered the EC50 of BETi to the submicromolar range [38]. Another BETi suggested to better block rapid growing tumour cells such as TNBC cells is MS645 [39].

Despite the different BETi discovered, the acquisition of resistance has hampered their clinical implementation for TNBC treatment [28,116]. Such a resistance seems to be caused by a decrease in the activity of the phosphatase tumour suppressor protein PP2A and consequent BRD4 hyperphosphorylation which, leads to a tighter binding to Estrogen receptor coactivator mediator subunit 1 (MED1) [28]. Then, MED1 activates transcription by the recruitment of non-bromodomain proteins [28]. Recent efforts using CRISPR-Cas9 and small-molecule inhibitor screens have identified synthetic lethal interactions between BETi and certain genes as well as resistance-responsible genes to BETi in TNBC such as *CDKN2A*, *ARID1A* and *TCEB3* [116]. BETi have also been considered for the indirect inhibition of MYC through the interference with *MYC*-dependent transcription via recruitment of the positive transcription elongation factor complex b (P-TEFb) after the observation of its recruitment by MYC [117] and its interaction with BRD4 [118,119].

Recently, four clinical trials have been planned to evaluate BETi in TNBC patients (Table 2). One clinical trial, NCT02698176, was a phase Ib dose exploration study with birabresib in participants with advanced solid tumours including TNBC. Although birabresib was found to be safe, the study terminated due to its limited efficacy. Other clinical trials are assessing the efficacy of BETi in combination with other agents. In particular, a phase II trial (NCT03901469) is currently studying the efficacy of another BETi, ZEN003694, in combination with the PARP inhibitor talazoparib. Moreover, anti-Programmed death-ligand 1 (PD-L1) antibodies have been administered with BETi in two other clinical trials: A phase I/IIa trial (NCT02419417) using the BETi BMS-986158 in combination with nivolumab with results still to be published [120], and a phase IIB trial, NCT03292172, using BETi RO6870810 in combination with atezolizumab. This latter trial was terminated in 2019 due to portfolio prioritisation [120].

### 3.2. Interference Peptides and Proteins

Interference peptides or proteins are therapeutic tools used to suppress the activity of TFs. They possess the same sequence of a native TF except for a few point mutations that led them inert but able to act as dominant negative by competing for the binding with its binding partners and the DNA. Among all the interference peptides/proteins developed, the development of OmoMYC is especially remarkable. OmoMYC is a 92-amino acids protein that harbours four-point mutations. These mutations prevent MYC molecular recognition [121]. After its successful inhibition of lung tumour growth in mice when administered intranasally and systemically [122], OmoMYC has just entered a phase I/II clinical trial (NCT04808362) supported by the spin-off Peptomyc S.L. The lead compound, OMO-103, is being evaluated for safety, pharmacokinetics and anti-tumour activity in 74 patients with advanced solid tumours including TNBC. 

Our laboratory has pioneered the development of functional penetrating ‘Phylomer’ peptide (FPPa) to efficiently deliver OmoMYC intracellularly and potentiate its anti-tumoral effect in vitro and in vivo in TNBC [20]. This could improve OmoMYC anti-tumoral performance in future clinical trials. Similarly, our previous works proved the potential of interference peptides against EN1, namely EN1-iPeps, to selectively decrease TNBC proliferation and sensitise TNBC cells to several chemotherapeutics [5]. Such a chemosensitisation power has also been demonstrated when EN1-iPeps were coupled to docetaxel nanoparticles in the TNBC syngeneic mice model T11 [40]. Further modification of the EN1-iPep by direct linkage with RGD peptides improved their cell selectivity and reduced tumour growth without observable toxicity [21]. SOX2 is another TFs with a relevant role in TNBC from which we have designed SOX2-iPeps. We have structurally characterised SOX2-iPeps and demonstrated their anti-tumoral activity in vitro in TNBC [49]. However, the effects in cell proliferation were mild. Regarding MZF1, interference peptides have been designed to disrupt the formation of the heterodimer MZF1/Elk-1 with a consequent change of the phenotype in TNBC cells. In particular, MZF-160–72 and Elk-1145–157 peptides fused to the cell penetrating peptide Transactivator of transcription (TAT) reduced the binding between MZF1 and Elk-1, cell migration and some EMT markers such as PKCα, Slug and Vimentin, and substantially increased E-Cadherin [43].

### 3.3. Peptidomimetics

A peptidomimetic is a molecule designed to mimic the structure and functionality of a natural peptide or protein; possess great stability and bioavailability, and has a pharmacological effect [123]. Peptidomimetics has recently classified in four different groups (Class A, B, C and D), depending on their degree of peptide character [123]. Among the peptidomimetics designed so far, there can be highlighted those that emulate p53 and have high affinity for its natural repressors MDM2 and/or MDMX. Some of them show anti-tumoral activity in vitro and in vivo, through the reactivation of the p53 pathway, and reached clinical trials [19,124]. Examples are SAH-p53-8, β3-peptide, DPMI-α and DPMI-γ, Pep-3, ATSP-7041 and ALRN-6924 [124,125,126].

In TNBC, the development of BC71 is remarkable. BC71 is a synthetic peptide that mimics the binding peptide of glucose-related protein 78 (GRP78), a protein that belongs to the family of heat-shock proteins, whose expression is up-regulated in response to stress. GRP78 is overexpressed in several human cancers including TNBC, and has been associated with chemoresistance, malignancy, and poor prognosis. The conjugation of BC71 to GRP78 induces apoptosis in murine TNBC 4T1 cells, through the activation of caspase-8, the induction of p53 and the inhibition of NFκB; also reduced the growth of 4T1 cell allografts [44]. Another protein targeted by peptidomimetics is SOC3S. Loss of SOCS3 expression is associated with cancer-associated inflammation and immunity suppression, favouring tumour growth and metastasis [45]. La Manna et al. designed the SOCS3 peptidomimetic KIRESS which is able to interact with the Janus kinase (JAK)/STAT/gp130 complex and inhibit tumour growth and lung metastases in TNBC MDA-MB-231 xenografts [45].

### 3.4. G-quadruplex Stabilisers

G-quadruplexes (G4s) are secondary DNA or RNA structures formed in G-rich sequences [127,128] that can regulate gene expression, especially of oncogenes. The discovery of molecules able to bind to such specific oncogene G4s has been very challenging. However, some reports have demonstrated their success. For instance, Yang et al. have identified several compounds able to interact and stabilise the hairpin containing G4 structure present in the negative strand of the MYCN gene from a 15,000 small molecules-containing microarray [129]. Another compound, CX-5461, with previous promising anti-tumoral activity against solid tumours [50], was found to act as G4 stabiliser and induce synthetic lethality in BRCA1/2 deficient TNBC cells and platinum-pretreated TNBC PDXs, with the precise mechanism yet to be elucidated [51]. Later, CX-5461 was shown to be synergistic at reducing cell proliferation, apoptosis, DNA damage and replication stress with the p53 activator APR-246 in TNBC cells [52]. In addition, in TNBC, Hu et al. designed and synthesised quinoxaline analogues to stabilise MYC promoter G4, known to repress MYC-dependent transcription. In particular, one of them, QN-1, showed the highest selectivity for MYC promoter G4 and decreased the growth of TNBC cells and 4T1 allografts [41]. A derivative of QN-1, described in this same work, was reported to also target topoisomerase 1 (Topo1). The dual targeting of MYC G4 and Topo1 resulted in an induction of DNA damage and a marked decrease in cell growth of TNBC cells and MDA-MB-231 xenografts [42].

### 3.5. Genome Engineering Tools

Another therapeutic possibility is to target TNBC oncogenic TFs at the gene level by using genome engineering tools. Suppression of SOX2 in BC has been possible with artificial zinc fingers proteins (ZFP) linked to epigenetic modifiers, such as DNA methyltransferase 3A (ZF-DNMT3A) designed to target the SOX2 promoter [46,48]. These constructs elicited long-lasting epigenetic silencing in vivo [46]. Moreover, the epigenetic modifier Super KRAB (Krüppel associated box) domain (SKD) linked to the same ZFP achieved SOX2 silencing in TNBC cells and MCF7 xenografts [47]. The ground-breaking genome engineering tool Clustered Regularly Interspaced Palindromic Repeats (CRISPR) has successfully suppressed ZEB1 in claudin-low TNBC cells and deciphered the mechanism of regulation of the theta-mediated end-joining (TMEJ) pathway [22].

## 4. TFs Used as Biomarkers of Stratification, Diagnosis and Prognosis in TNBC

Not only TF can be considered promising targets for TNBC therapy, but they can also be useful for classification and prognosis prediction.

In 2011, Lehmann et al. subclassified TNBC tumours by taking into account 21 public microarray databases [130]. One of the subtypes identified was the luminal androgen receptor (LAR) subtype, characterised by an activated AR signalling. Patients with LAR TNBC showed a significant decrease in disease-free survival (DFS), and higher overall survival (OS) compared to other TNBC subtypes [130,131,132]. AR loss is a predictor of early recurrence in TNBC [130,131]. More recently, a new category that conforms 57–90% of TNBC has emerged, the quadruple negative BCs (QNBC), characterised by the absence of AR besides ER, PR and HER2 [57]. These cancers have poorer prognosis and survival outcomes than TNBC, even with the administration of adjuvant chemotherapy [133]. However, such clinical outcomes partially depend on the patients’ geographical ancestry [134].

BRD4 was found to be significantly higher expressed in basal-like BCs compared to luminal breast tumours [116] even within TNBC, suggesting a relevant role of BRD4 in BCs with basal-like phenotype. The same authors discovered that lower BRD4 expression was correlated with shorter DFS [116]. Conversely, the study noted that high levels of BRD4 were correlated with unfavourable prognosis and shorter (OS) and (PFS) [59]. Moreover, BRD4 expression positively correlated with a major tumour infiltration of B cells, CDT4+ T cells, CDT8+ T cells, macrophages, neutrophils and dendritic cells [59]. Phosphorylation of BRD4 (pBRD4) is another interesting biomarker. It has significantly been associated with relapse in a cohort of 132 TNBC patients [135], where predicted poor outcome in terms of OS and DFS [135].

FOXC1 overexpression has been associated with more aggressive tumour behaviour and poorer prognosis [65]. In addition, FOXC1 has been suggested to predict poor prognosis in TNBC together with miR-135b-5p, miR-9-3p, miR-135b-3p and miR-455-5p [62]. Notably, FOXC1 expression is interrogated in the prediction analysis of microarray 50 (PAM50) assay, a BC profiling tool for the four major intrinsic subtypes and predictor of recurrence risk. High FOXC1 expression levels correlate with the basal-like BC subtype and higher recurrence risk. Regarding metastasis, FOXC1 expression has been correlated with a higher incidence of brain and lung metastases, and a decrease in metastasis-free survival [136].

Elevated MYC signalling has significantly been associated with shorter DFS in BC [137] and higher MYC expression significantly correlated with shorter OS in TNBC [138]. Interestingly, the least responding tumours to neoadjuvant therapy presented an elevated MYC signature and earlier disease recurrence compared to the most responding tumours [137]. As FOXC1, MYC conforms the gene signature of the PAM50 assay and positively correlates with the basal-like subtype and higher recurrence risk.

Regarding EN1, cytoplasmic EN1 expression has been associated with higher OS rate unlike *EN1* mRNA expression which did not correlate with any particular clinical outcome [72]. These results suggest that this TF exert different effects on patient survival depending on its localization. In another study, high expression of EN1 correlates with short OS and increased risk of developing brain metastasis in TNBC patients due to the expression and dependency of these tumours for neural survival factors [6]. 

Basal-like BCs or TNBC show overexpression of SOX members such as SOX2, SOX9, SOX10 and SOX11 [89,139,140,141]. Expression of SOX9 and SOX10 have been correlated to shorter OS [139,140,141]. In contrast, SOX10 expression failed to predict prognosis in terms of DFS, distant DFS and OS [142]. SOX8 was found to be expressed in 44% of TNBC samples (out of 250 samples) and was positively and significantly associated with tumour size and stage [143]. Additionally, high SOX8 expression significantly correlated with shorter DFS and OS in TNBC patients [143].

Regarding ZEB1, its expression has been assumed to predict poor clinical outcome in TNBC in terms of OS and DFS [144,145]. It has also been associated with a more aggressive phenotype [146] and tendency to metastasise both in the lymph nodes and distantly [144]. ZEB1 together SOX10 and MZF1, were identified as part of a TF activation TNBC oncogenic core by computational analysis and posteriorly by functional assays [78]. Interestingly, tumours presenting this activation gene signature resembled TNBC disease and had the worst prognosis, indicating that the presence of these TFs could be useful for TNBC prognosis re-classification [78]. Moreover, expression of ZEB1 and the long non-coding RNA (lncRNA) linc-ZNF469-3 were correlated with TNBC tumour recurrence [96]. Similarly, both high ZEB1 levels and SOX8 correlated positively in a cohort of 250 TNBC samples, and were significantly associated with shorter DFS and OS [143], possibly due to the implication of the ZEB1-SOX2 axis in the regulation of the CSC population [143].

p53 accumulation has been associated with steroid hormone receptor-negativity and high-grade tumours with high metastatic potential [107]. Its presence and mutated variants have been correlated with worse prognosis in TNBC [147]. Moreover, patients with positive p53 had significantly shorter OS and DFS [147]; therefore, this biomarker could be useful for TNBC stratification into different prognosis and aggressivity. The basal-like 1 or BL1 subtype of Lehmann’s classification [130] was characterised by mutations in DNA damage response genes such as *TP53* (92% of the cases). Interestingly, this particular subtype showed the highest pathologic complete response rate to cisplatin and taxane-based neoadjuvant chemotherapy [148].

HMGA1 expression positively correlated with tumour aggressiveness in TNBC [111]. Moreover, high HMGA1 expression was significantly associated with shorter OS, relapse free survival, distant metastasis free survival and post-progression survival in BC. At the same time, among all PAM50 subtypes, basal-like BCs were the ones showing the highest expression of HMGA1 [111]. Altogether highlights a crucial role of HMGA1 in the progression of TNBC and its great value as prognosis predictor in these tumours.

## 5. Future Perspectives

TFs comprise 20% of all oncoproteins [149] and their activity has been found to be altered across many cancer types. They are final direct effectors of oncogenic signalling pathways leading to any of the hallmarks of cancer [150], and their therapeutic inhibition is, therefore, very attractive. Many efforts have been done to disrupt protein–protein and protein–DNA interactions as well as genetically blocking the expression of TFs. Additionally, other strategies designed to inhibit the activity of TFs could also result promising. Posttranscriptional modifications have been shown to modulate TFs activity. These include phosphorylation in serine, threonine or tyrosine; methylation in lysine or arginine; acetylation in lysine; ubiquitination, SUMOylation; and ADP ribosylation [150]. The activity of Runt related TF 1 (RUNX1), a TFs responsible of poor prognosis and aggressivity in TNBC [151,152], can be modified by phosphorylation, methylation and acetylation [153]. However, the effect of such RUNX1 posttranscriptional modifications in TNBC growth has not yet been explored, and the modulation of RUNX1 activity by eligible enzymes represents a possible therapeutic approach. Nevertheless, the utilisation of enzymes can lead to significant off-target effects due to their ubiquitous function. Another interesting strategy that can be used for blocking TFs are antibodies as they can virtually target any given oncoprotein. Whilst the use of antibodies was inconceivable in the past for targeting intracellular targets due to their inability to cross biological membranes, it seems plausible now with the use of nanotechnology [154]. Deng et al. delivered monoclonal antibodies anti-S100A4 in the cytoplasm using liposomes. Such a therapeutic agent reduced in vivo tumour growth in TNBC 4T1 allografts [155]. Currently, there are not succeeded studies capable of inhibit TFs with this approach, however, it holds enormous promise.

Another emerging and very promising approach to block the activity of TFs is the utilisation of targeted protein degradation using small organic molecules called degraders. These molecules facilitate the degradation of the target protein via proteasome by interacting directly with the protein, by interacting with E3 ubiquitin ligases or by crosslinking both [156]. Examples of the latter type of degraders are proteolysis targeting chimeras (PROTACs) and specific and non-genetic IAP-dependent protein erasers (SNIPERS). Interestingly, some of them have reached clinical trials such as the PROTACs ARV-110, against AR in prostate cancer patients, [157] and ARV-471, against ER in ER+/HER- BC patients. Recently, transcription factor targeting chimeras (TRAFTACs) have been specifically designed to target the degradation of the oncogenic TFs NF-κB and Brachyury [158]. Preclinically in TNBC, Metformin has been shown to be effective at targeting the degradation of the TF Krüppel-like factor 5 (KLF5), which decreased the stem cell population [159]. In addition, a recruiting molecule was successful at degrading the TF FOXM1 and exerted in vivo anti-tumoral activity [160]. Moreover, two PROTACs against BRD4, MZ1 and ARV-825, reduced tumour growth in BETi-resistant TNBC models in vitro and in vivo [161]. Although the development of degraders and their exploration in TNBC are still in its infancy, they certainly hold great potential.

## Figures and Tables

**Table 1:** TFs relevant for the onset and progression of triple negative breast cancers. For each TF there are indicated the therapeutic intervention, breast cancer model used, dose, route of administration, and related references.

Preclinical Studies						
TF	Intervention	Breast Cancer Model	In Vitro/In Vivo	Dose	In Vivo Mode of Administration	Ref
AR	Small molecule inhibitor Enzalutamide	TNBC lines: SUM159PT, HCC1806, BT549, and MDA-MB-231. TNBC xenografts: SUM159PT and HCC1806	Yes/ Yes	Cells: 10 μM Xenografts: 50 mg/Kg daily dose	Orally (food chow)	[23]
		TNBC cell lines: SUM159PT, BT549 and MDA-MB-453 TNBC xenografts: SUM159PT	Yes/ Yes	Cells: 10 μM Xenografts: 20 μM	Pretreatment of injected cells and orally (food chow)	[24]
BRD4	Small molecule inhibitors JQ1 and MS417	TNBC cell lines: SUM1315, BT549, MDA-MB-157, SUM129 and Hs578T Xenograft: SUM1315	Yes/ Yes	Cells: JQ1 1 μM Xenografts: JQ1 50 mg/Kg; MS417 20 mg/Kg	Not mentioned	[25]
	Small molecule inhibitor JQ1	TNBC cell lines: MDA-MB-231, SUM149PT; BC cell lines: MCF-7 and T47D	Yes/ No	Cells: 400 nM	N/A	[26]
	Small molecule inhibitor JQ1	TNBC cell lines: MDA-MB-231; BC cell lines: T47D and SK-BR-3; normal cell line: MCF-10A Xenografts: MDA-MB-231 and T47D	Yes/ Yes	Cells: JQ1 500 nM	N/A	[27]
	Small molecule inhibitor JQ1	TNBC cell lines: SUM149, and SUM159; 40 BC cell lines TNBC Patient derived xenograft: IDC50	Yes/ Yes	Cells: JQ1 0.5–20 μM Xenografts: JQ1 50 mg/kg daily dose	Not mentioned	[28]
	Small molecule inhibitor JQ1	TNBC cell lines: MDA-MB-231, HCC38, BT549, HCC1143, HCC70, and MDA-MB-468	Yes/ No	Cells: JQ1 0.5 μM	N/A	[29]
	Small molecule inhibitors JQ1 and GSK525762A	TNBC cell lines: MDA-MB-157, MDA-MB-231 and BT-549	Yes/ No	Cells: JQ1 0.5–5 μM and SK525762 0.5–5 μM	N/A	[30]
	Small molecule inhibitors: JQ1 and VS-6063	16 TNBC cell lines Xenograft: Orthotopic 4T1	Yes/ Yes	Cells: JQ1 0.5–1 μM and VS-6063 0.5–10 μM. Xenografts: JQ1 25 mg/Kg and VS-6063 50–75 mg/Kg	Oral gavage	[31]
	JQ1-loaded polydopamine nanoplatform (PDMN-JQ1) + 808 nm laser irradiation	TNBC cell lines: 4T1 TNBC allograft: 4T1	Yes/ Yes	Cells: PDMN-JQ1 up to 200 μg/mL with 808 nm irradiation Allografts: PDMN-JQ1 200 μg/mL and then irradiated at 808 nm, 1 W/cm^2^, 300 s	IT	[32]
	Small molecule inhibitors JQ1 and enzalutamide	TNBC cell lines: MDA-MB-231, MDA-MB-453, MDA-MB-468 and BT-20 Xenograft: MDA-MB-231	Yes/ Yes	Cells: 0.1–50 μM (+)-JQ1 and enzalutamide Xenografts: JQ1 50 mg/kg, enzalutamide 30 mg/kg, and the combination	IP and oral gavage	[33]
	Small molecule inhibitor JQ1	TNBC cell lines: HCC1143, MDA-MB-468, HCC70, MDA-MB-231, BT549, HCC38 and MDA-MB-453 TNBC xenografts: MDA-MB-231 and MDA-MB-468 Patient-derived xenograft: BCM-4013	Yes/ Yes	Cells: JQ1, I-BET151, I-BET762 up to 1000 nM Xenografts: JQ1 50 mg/kg	IP	[34]
	Small molecule inhibitors JQ1, OTX015 and CPI-637	TNBC cell lines: MDA-MB-231, MDA-MB-468, SUM-149, HCC1806, WHIM2 and WHIM12	Yes/ No	Cells: Up to 10 μM	N/A	[35]
	Small molecule inhibitors INCB054329 and JQ1	TNBC cell lines: MDA-MB-468 and CAL-51 and clonally derived cell lines TNBC patient-derived xenografts: TNBC PDX TM00096, TM00090, TM01273, BCM-2147 and HBCx1	Yes/ Yes	Cells: Up to 1 μM Xenografts: JQ1 50 mg/kg; INCB054329 50 mg/kg	Orogastric gavage	[36]
	Small molecule inhibitor OTX015	TNBC cell lines: HCC1937, MDA-MB-231 and MDA-MB-468 TNBC xenografts: MDA-MB-231	Yes/ Yes	Cells: Up to 650 nM Xenografts: 50 mg/Kg	IP	[37]
	Small molecule inhibitor JQ1, CPI-203, I-BET151, and I-BET76	TNBC cell lines: MDA-MB-231, BT-549 and HCC1937 TNBC xenografts: MDA-MB-231	Yes/ Yes	Cells: Up to 1 μM Xenografts: JQ1 30 and 50 mg/Kg	IP	[38]
	Small molecule inhibitors MS645, MS660, MS688, and JQ1	TNBC cell lines: MDA-MB-231, Hs578T, HCC1806, SUM1315, BT549 and HCC38; normal cell lines: RAW264.1 and MCF-10A	Yes/ No	Cells: Up to 100 μM	N/A	[39]
EN1	Interference peptide EN1-iPep	TNBC cell line: SUM149PT, SUM159PT, SUM102PT, MDA-MB-468, HC1806, SUM229, MDA-B-435s, MDA-MB-453 and MDA-MB-231; BC cell lines: BT-474, SKBR3, T47D and MCF-7; normal cell line: HUMEC	Yes/ No	Cells: Up to 100 μM	N/A	[5]
	Docetaxel nanoparticles coated with the interference peptide EN1-iPep	TNBC cell lines: SUM149 and T11; normal cell line: MCF-10A TNBC allograft: T11	Yes/ Yes	Cells: Up to nanoparticles 1 mg/mL + EN1-iPep 60.25 μM Allografts: nanoparticles 2.5 mg + EN1-iPep 0.5 mg	IT	[40]
	Docetaxel nanoparticles coated with the interference peptide EN1-RGD1-iPep	TNBC cell lines: SUM149, SUM159 and T11; normal cells: NIH/3T3 and MCF-10A TNBC allograft: T11		Cells: Up to nanoparticles 1.7 mg/mL + EN1-RGD1-iPep 114.8 μM Allografts: nanoparticles 0.5 mg + EN1-iPep 0.5 mg	IV	[21]
MYC	iPep against MYC (FPPa-OmoMYC)	NIH-3T3, HDEF, MCF-7, ZR-751, MDA-MB-231, MCF-10A, MCF-12A, SUM149, and SUM159 TNBC allograft: T11	Yes/ Yes	Cell lines: Up to 15 μM Allograft: 32.2 mg/Kg	IT	[20]
	DNA G-quadruplex stabiliser QN-1	TNBC cell lines: 4T1 Allograft: 4T1	Yes/ Yes	Cell lines: Up to 10 μM Allografts: 2.5, 5 and 10 mg/Kg	IP	[41]
	DNA G-quadruplex stabilisers derivative of QN-1	TNBC cell lines: MDA-MB-231 Xenografts: MDA-MB-231	Yes/ Yes	Cell lines: Compound 5 up to 20 μM Xenografts: compound 5 2.5 and 5 mg/Kg	IP	[42]
MZF1	Interference peptides TAT-MZF-1_60–72_ and TAT-Elk-1_145–157_	TNBC cell lines: Hs578T, MDA-MB-231, and MDA-MB-468; BC cell lines: MCF-7; others: HEK-293 Xenografts: MDA-MB-231 and Hs578T cells	Yes/ Yes	Cell lines: Up to 100 μM Xenografts: N/A	SC injection of genetically engineered cells with MZF1_60–72_	[43]
GRP78	Peptidomimetic BC71	TNBC cell lines: 4T1; normal cell lines: HUVEC Allograft: 4T1	Yes/ Yes	Cell lines: 100 μM Allograft: 250 μg	IV	[44]
SOCS3	Peptidomimetic KIRESS	TNBC cell lines: MDA-MB-231 and 4T1 Allograft: 4T1; xenograft: MDA-MB-231	Yes/ Yes	Cell lines: 10 μM Allograft: 10 mg/Kg	IP	[45]
SOX2	Genome engineering tools: Zinc finger proteins ZF598-DNMT3A and ZF598-SKD	BC cell lines: MCF-7 Xenografts: MCF-7	Yes/ Yes	Cell lines: transfected for stably expressing ZF598-DNMT3A and ZF598-SKD Xenograft: N/A	SC injection of genetically engineered cells with ZF598-DNMT3A	[46]
	Genome editing tool: Zing finger proteins ZF-552SKD, ZF-598SKD, ZF-619SKD and ZF-4203SKD	TNBC cell lines: MDA-MB-231, MDA-MB-435s, MDA-MB-468, BT549, SUM102, SUM149, SUM159 and MDA-MB-453; BC cell lines: MCF-7, SK-BR-3 and ZR-75-1; normal cell lines: MCF-12A Xenografts: MCF-7	Yes/ Yes	Cell lines: transfected for stably expressing ZF598-DNMT3A and ZF598-SKD Xenograft: N/A	SC injection of genetically engineered cells with ZFP-ATF	[47]
	Genome editing tool: Zing finger proteins ZF-97-SKD/DNMT3A, and ZF-126 SKD/DNMT3A and ZF-452 SKD/DNMT3A	BC cell line: MCF-7 cells with stable expression of ZF-552 DNMT3a	Yes/ No	Cell lines: transfected for stably expressing the ZFs	N/A	[48]
	Interference peptide SOX2-iPep	TNBC cell line: T11; BC cell line: MCF-7; normal cell line: HDEF	Yes/ No	Cell lines: Up to 100 μM	N/A	[49]
ZEB1	Genome editing tool CRISPR/Cas9	TNBC cell lines: BT-20, MDA-MB-468, BT549, SUM159, MDA-MB-231; Hs578T and CAL-120; BC cell lines: HCC70, HCC1937, Normal cell lines: HMEC	Yes/ No	Cell lines: Lentivirally transfected for stably expressing the CRISPR/9 constructs	N/A	[22]
Others or undetermined	DNA G-quadruplex stabiliser CX-5461	A panel of TNBC cell lines	Yes/ Yes	Cells: IC50 in the nanomolar range Xenografts: not in TNBC	N/A (not in TNBC)	[50]
		TNBC cell lines: BT20, CAL51, HCC1806, HCC1395, MDA-MB-436, MDA-MB-468 and HCC38 TNBC PDXs: CTG-1019, CTG-0012, CTG-0888, CFIB-NB02 and CFIB-70620	Yes/ Yes	Cells: IC50 ≤ 10^−7^ M Xenografts: 50 mg/Kg	Oral gavage	[51]
		TNBC cell lines: MDA-MB-231, BT549 and SUM159PT; BC cell lines: MCF-7, T47D, MDA-MB-361, MDA-MB-453, SK-BR-s3, BT474, BT483	Yes/ No	Cells: Up to 2 μM	N/A	[52]

**Table 2:** Clinical studies using different inhibitors of TFs in TNBC patients. For each TF there are indicated the drug used, the design and participants, the primary outcomes, the status and main results, the identification number of the clinical trial, and the related reference.

Clinical Trials					
TF	Drugs	Design/Participants	Primary Outcomes	Status and Main Results	Reference Clinical Trial ID
AR	Abiraterone and prenidsone	Phase II 34 Locally advanced or mTNBC patients	CBR, ORR, PFS, OS, duration of response and safety	Completed. Abiraterone plus prednisone treatment is beneficial for some patients with molecular apocrine tumours	[53] NCT01842321
	Enzalutamide	Phase II 118 TNBC patients	CBR, ORR, PFS, adverse effects	Completed. Enzalutamide demonstrated clinical activity and was well tolerated	[54] NCT01889238
	Seviteronel	Phase I 19 locally advanced or mTNBC	CBR, safety, tolerability and maximum tolerated dose	Completed. Seviteronel was generally well tolerated but not ORR were provided	[55] NIHMS966623
BRD4	Birabresib	Phase IB 13 advanced solid tumours including TNBC	Dose limiting toxicity	Terminated	NCT02698176
	ZEN003694 and talazoparib	Phase II 49 TNBC patients	Related adverse events, pharmacokinetics, ORR, PR, SD and PFS	Under evaluation	NCT03901469
	R06870810 and atezolizumab	Phase IIB 36 ovarian or TNBC	DLT, pharmacokinetics, OR, OS, PFS	Terminated	NCT03292172
	BMS-986158 and/or nivolumab	Phase I/IIA 417 advanced solid tumours including TBNC	Adverse effects, pharmacokinetics, ORR	Completed. Results not yet posted	NCT02419417
MYC	OmoMYC	Phase I/II 74 advanced solid tumours	Adverse effects, pharmacokinetics, ORR	Recruiting	NCT04808362

Abbreviators: BC: breast cancer; CBR: clinical benefit rate; DLT: dose limiting toxicity; IP: intraperitoneally; IT: intratumourally; IV: intravenously; mBC: metastatic breast cancer; ORR: objective response rate; OS: overall survival; PDX: patient-derived xenograft; PFS: progression free survival; PR: partial response; Ref: reference; SC: subcutaneous; TF: transcription factor.
